# Supplementary material for: Content-rich biological network constructed by mining PubMed abstracts
Source: BMC Bioinformatics. 2004 Oct 8;5:147. doi: 10.1186/1471-2105-5-147 (PMC528731; doi:10.1186/1471-2105-5-147)
Supplement: Additional File 2 — The original results of the above study (non-essential files are deleted to keep the file size under the limit set by BMC bioinformatics). [file 1471-2105-5-147-S2.bz2 › chilibotAdditionalFile2/dip40/122ID8668348E466/html/MAP2K1_BRAF.html]

 


 **MAP2K1** and **BRAF** 
  
Found 75 abstracts in PubMed, retrieved 40.  
 

 What does Google say? 
 PDF only 
| .edu only 

---

**Interactive relationship** (e.g. stimulation, inhibition, etc)

**Inhibitory relationship**- A dominant negative mutant of B Raf  [ **BRAF** ] , but not of Raf 1, blocked the cAMP induced activation of ERK, indicating that B Raf  [ **BRAF** ]  is the MEK 1  [ **MAP2K1** ]  upstream regulator mediating this cAMP effect.  Ref: 10856235 EMBO J, 2000
- We found that 8 bromoadenosine cyclic monophosphate 8Br cAMP induced the activation of ERK and mitogen activated protein kinase MEK  [ **MAP2K1** ] , but inhibited B Raf  [ **BRAF** ]  kinase via a protein kinase A PKA mediated mechanism.  Ref: 12479852 Leuk Res, 2003
- The activity of MEK  [ **MAP2K1** ]  is essential for cell survival mediated by B Raf  [ **BRAF** ]  overexpression, since either treatment with the specific MEK  [ **MAP2K1** ]  inhibitor PD98059 or expression of a dominant inhibitory MEK  [ **MAP2K1** ]  mutant blocks the antiapoptotic activity of B Raf  [ **BRAF** ] .  Ref: 10409722 Mol Cell Biol, 1999
- B Raf  [ **BRAF** ]  inhibits programmed cell death downstream of cytochrome c release from mitochondria by activating the MEK  [ **MAP2K1** ]  Erk pathway.  Ref: 10409722 Mol Cell Biol, 1999
**Neutral relationship**- In contrast, B Raf  [ **BRAF** ] , a MAPKKK specific for the MEK  [ **MAP2K1** ]  ERK module, binds PA28gamma and a.  Ref: 12650640 Biochem J< MedlineTA>Biochem J, 1999
- We have recently established Rat 1 fibroblast cell lines overexpressing B Raf  [ **BRAF** ] , leading to activation of the MEK  [ **MAP2K1** ]  Erk mitogen activated protein kinase pathway.  Ref: 10409722 Mol Cell Biol, 1999
- Raf comprises a family of three kinases, A Raf, B Raf  [ **BRAF** ]  and Raf 1, which are best known as key regulators of the MEK  [ **MAP2K1** ]  MAPK ERK cascade.  Ref: 11950876 J Cell Sci, 2002
- We failed to observe any significant induction and or modulation of the A Raf, B Raf  [ **BRAF** ]  or c Raf 1 kinase activities, thus suggesting the existence of a MEK  [ **MAP2K1** ]  kinase different from the classical Raf kinases that directly or indirectly can be modulated by Ca2 calmodulin.  Ref: 9867813 J Biol Chem, 1999
- MEK  [ **MAP2K1** ]  ERK activation is normal in Raf 1 deficient cells and embryos, and is probably mediated by B RAF  [ **BRAF** ] .  Ref: 11296228 EMBO J, 2001
- Together, these data define a novel signaling pathway for M 1 mAChR, where increases in Ca 2 and diacylglycerol stimulate the sequential activation of CalDAG GEFI, Rap1, and B Raf  [ **BRAF** ] , resulting in the activation of MEK  [ **MAP2K1** ]  and ERK1 2.  Ref: 11292831 J Biol Chem, 2001

**Non-interactive relationship** (e.g. studied together, co-existance, homology, etc.)

- Down stream of PKA, the activity of B Raf  [ **BRAF** ]  was significantly decreased by nicotine in SH SY5Y cells, as determined by direct measurement of MEK1  [ **MAP2K1** ]  phosphorylation or in vitro kinase assays.  Ref: 11905997 J Neurochem, 2002
- Interestingly, B Raf  [ **BRAF** ]  and Raf 1 activity towards MEK  [ **MAP2K1** ]  as measured by the immunoprecipitation kinase cascade assay are both significantly increased in the A Raf deficient MEFs.  Ref: 11821947 Oncogene, 2002
- After immunoprecipitation of Raf 1 and B Raf  [ **BRAF** ] , the basal glutathione S transferase MEK1  [ **MAP2K1** ]  phosphorylation observed in resting platelets was not upregulated by thrombin and was still observed in the absence of anti Raf 1 or anti B Raf  [ **BRAF** ]  antibodies.  Ref: 12435596 FEBS Lett, 2002
- To investigate the importance of Raf MEK  [ **MAP2K1** ]  ERK signaling in human hematopoietic cell growth, the effects of three different Raf genes, A Raf, B Raf  [ **BRAF** ]  and Raf 1, on cell cycle progression and regulatory gene expression were examined in TF 1 cells transformed to grow in response to beta estradiol regulated DeltaRaf ER genes.  Ref: 12429936 Cell Cycle< MedlineTA>Cell Cycle, 2002
- It thus appears that the B Raf  [ **BRAF** ]  MEK  [ **MAP2K1** ]  Erk pathway confers protection against apoptosis at the level of cytosolic caspase activation, downstream of the release of cytochrome c from mitochondria.  Ref: 10409722 Mol Cell Biol, 1999
- 17beta Estradiol increased B Raf  [ **BRAF** ]  activity and MEK  [ **MAP2K1** ]  MAP kinase ERK kinase dependent ERK phosphorylation in cerebral cortical explants derived from both ERKO and their wild type littermates.  Ref: 10684871 J Neurosci, 2000
- Surprisingly, in contrast to proliferative cells, we found that the serine threonine kinases Raf 1 and B Raf  [ **BRAF** ]  were not an intermediate kinase between conventional PKCs and MEK1  [ **MAP2K1** ]  2.  Ref: 12435596 FEBS Lett, 2002
- the frequency of transformation was dependent on the particular oncogene examined, as more factor independent cells were isolated after infection with activated retroviruses encoding A Raf or Raf 1 than were with MEK1  [ **MAP2K1** ]  or B Raf  [ **BRAF** ] .  Ref: 11531015 Cancer Detect Prev, 2001
- Fractions containing kinase activity were also probed with antibodies against MEK  [ **MAP2K1** ]  kinase 1, Raf 1, A Raf, B Raf  [ **BRAF** ] , Mos, and Tpl 2.  Ref: 10226075 Am J Respir Cell Mol Biol, 1999
- Because c Raf activation and MEK  [ **MAP2K1** ]  activation were inversely affected by PI3 K and cAMP dependent pathways, we examined whether IL 3 activated the alternative Raf isoforms A Raf and B Raf  [ **BRAF** ] .  Ref: 10066754 J Biol Chem, 1999
- 17 beta oestradiol increased B Raf  [ **BRAF** ]  activity and MEK  [ **MAP2K1** ]  dependent ERK phosphorylation in explants of wild type and ERKO cerebral cortex.  Ref: 10965502 Novartis Found Symp, 2000
- GLP1 stimulated activation of Erk is blocked by inhibitors of MEK  [ **MAP2K1** ] , but GLP1 does not induce the activation of A Raf, B Raf  [ **BRAF** ] , C Raf, or Ras.  Ref: 12364324 J Biol Chem, 2002
- We found that H. pylori infection of gastric epithelial cells activated the MEK1  [ **MAP2K1** ]  2 ERK1 2 cascade through cAMP dependent stimulation of Rap1 and B Raf  [ **BRAF** ] , but not Ras c Raf 1, leading to potent transactivation of the human HDC promoter.  Ref: 11790728 FASEB J, 2002
- Furthermore, ATP did not activate B Raf  [ **BRAF** ] , the major isoform of Raf in the brain, nor other Mek  [ **MAP2K1** ]  activators such as Mek  [ **MAP2K1** ]  kinase 1 MekK1 and MekK2 3.  Ref: 10696092 Br J Pharmacol, 2000
- Disruption of the Raf MEK  [ **MAP2K1** ]  ERK MAPK kinase pathway, either by RAS or  **BRAF**  mutation, was detected in approximately 62% of all CC and is therefore one of the most frequent defects in cholangiocellular carcinogenesis.  Ref: 12692057 Gut, 2003
- Activation of MEK  [ **MAP2K1** ]  is not only necessary but also sufficient for cell survival because overexpression of constitutively activated MEK  [ **MAP2K1** ] , Ras, or Raf 1, like B Raf  [ **BRAF** ] , prevents apoptosis after growth factor deprivation.  Ref: 10409722 Mol Cell Biol, 1999
- Thus, our studies demonstrate for the first time to our knowledge that the GalphaS >cAMP >Rap1 >B Raf  [ **BRAF** ]  >MEK1  [ **MAP2K1** ]  2 >ERK1 2 pathway is critical for H. pylori dependent epithelial gene regulation, which can be induced via a bioactive component apart from the site of bacterial colonization.  Ref: 11790728 FASEB J, 2002
- Taken together, our results identify a novel mechanism whereby a Gi o protein coupled receptor inhibits cell proliferation by stimulating ERK signaling via a SHP 1 SHP 2 PI3K Ras Rap1 B Raf  [ **BRAF** ]  MEK  [ **MAP2K1** ]  pathway.  Ref: 12878607 J Biol ChemJ Biol Chem, 2002
